# Inter-letter spacing, inter-word spacing, and font with dyslexia-friendly features: testing text readability in people with and without dyslexia

**DOI:** 10.1007/s11881-020-00194-x

**Published:** 2020-03-14

**Authors:** Jessica Galliussi, Luciano Perondi, Giuseppe Chia, Walter Gerbino, Paolo Bernardis

**Affiliations:** 1 Department of Life Sciences, University of Trieste, Trieste, Italy; 2 IUAV University of Venice, Venice, Italy; 3 CHIALAB, Bologna, Italy

**Keywords:** Dyslexia, EasyReading, Font, Letter spacing, Reading

## Abstract

Over the last years, several studies have suggested a possible link between dyslexia and deficits in low-level visual processing (e.g., excessive crowding). At the same time, specially designed “dyslexia-friendly” fonts appeared on the market. This class of fonts presents two main features: the particular graphic characteristics of the letterform designed to avoid confusion between similarly shaped letters, and wider inter-letter and inter-word spacing to limit crowding. The literature testing the efficacy of “dyslexia-friendly” fonts in improving reading accuracy and increasing reading speed is controversial. We evaluated the impact of letterform (with vs. without dyslexia-friendly graphic features), inter-letter spacing (standard vs. increased), and inter-word spacing (standard vs. increased) on reading accuracy and speed. Two groups of 64 children each, with and without dyslexia, read aloud 8 equivalent texts. The data collected failed to show any effect from the letterform. As regards spacing, the data showed that reading speed is impaired by an increase in inter-letter spacing not combined with an adequate increase in inter-word spacing.

**Electronic supplementary material:**

The online version of this article (10.1007/s11881-020-00194-x) contains supplementary material, which is available to authorized users.

Studies on developmental dyslexia (DD) in English-speaking countries show that prevalence ranges from 5 to 17.5% (Demonet, Taylor, & Chaix, 2004; Shaywitz, Fletcher, & Shaywitz, 1994). A recent study conducted in Italy by Barbiero et al. (2019) showed a prevalence of 3.5%. One of the reasons for this wide range in prevalence is the different orthographic complexity of various languages, which exacerbates some symptoms of dyslexia (Landerl et al., 2013).

Traditional approaches treat DD as a phonological processing impairment based on difficulty integrating letters and speech sounds. New theories suggest that phonological impairment cannot explain all deficits reported in people with dyslexia; indeed, even impaired low-level visual processing can produce dyslexia. Gori and Facoetti (2015) suggested a possible link between visual crowding—difficulty in identifying items when the items in question are surrounded by many others—and dyslexia. This link is supported by data from Joo, White, Strodtman, and Yeatman (2018), who identified a subgroup of individuals with dyslexia and elevated crowding. This group of people with dyslexia read faster when a text is rendered with increased inter-letter and inter-word spacing.

Over the last years, several studies have investigated how to alleviate deficits in the visuospatial processing of letters and words. In particular, whether it is possible to improve reading in people with dyslexia using dedicated fonts.^1^ This specific class of typefaces is called dyslexia friendly (DF). DF fonts are thought to help people with dyslexia to recognize letters, distinguish between letters of similar shapes, and limit crowding effects. Indeed, the facilitating characteristics are a specific letterform (i.e., increased thickness near the bottom; angling and changing the height and the contours of similarly shaped letters; serif or sans serif types) to prevent reversals, rotations, and misordering, and an increased spacing to limit crowding.

Research investigating the relevance and impact of DF fonts on reading abilities focused mainly on these two aspects: the graphic characteristics of the letterform and/or spacing. Some of these studies focused only on the space between letters and words. In the most common fonts, such as Times New Roman (TNR) Regular or Arial Regular, the regular space between two lowercase letters may vary roughly from 0 to 15% of the body size, while the regular space between two words corresponds approximately to 20–25% of the body size. The seminal work of Zorzi et al. (2012) showed that an extra-large space (TNR Regular inter-letter spacing enlarged by 2.5 pt. on 14 pt. body size, + ~ 18% of the body size) improves reading for children with dyslexia. Unfortunately, the authors did not match the length of the compared sentences; in fact, it has been acknowledged that shorter lines facilitate reading (Schneps et al., 2013). The results of Zorzi were partly replicated by Hakvoort, van den Boer, Leenaars, Bos, and Tijms (2017) who, using sentences of the same length, found that an extra-large inter-letter spacing decreases the number of errors significantly, but does not increase reading speed in children with dyslexia.

Other studies have focused on the graphic features of both the letterform and spacing. Bachmann (2013) tested a specific font (EasyReading^™^) developed for readers with dyslexia, which integrates particular graphic features (e.g., letterform with dedicated serifs, and longer ascenders and descenders) and enlarged inter-letter and inter-word spacing (spacing between two lowercase letters vary between 16 and 18% of the body size, and spacing between two words corresponds to 39% of the body size; see Bachmann and Mengheri (2018) for an English translation of the study). Bachmann’s results showed that children with dyslexia read an EasyReading^™^ text faster and with less error when compared with a Times New Roman text.

Unfortunately, Bachmann’s study did not provide any information on the relative contribution of letterform and spacing on the final effect. Marinus et al. (2016) dissociated the effect of these two variables comparing the DF font Dyslexie with Arial Regular, and with a version of Arial having the same spacing as Dyslexie. Their results clearly showed that it is possible to obtain the same facilitatory effect obtained with Dyslexie on sentence reading, even when Arial has the same spacing as Dyslexie, and that a dedicated letterform is not necessary. This “increased spacing” effect has partially been replicated by Duranovic, Senka, and Babic-Gavric (2018), who found an improvement in accuracy, but not in reading speed, in a group of children with dyslexia, all native Bosnian speakers.

Kuster et al. (2017, Experiment 1) tested a large group of 170 children with dyslexia with the DF font Dyslexie, without finding evidence of a dedicated letterform effect. In particular, they focused on Dyslexie, Arial Regular, and TNR Regular—like the authors of previous studies—but did not find any increased spacing effect. Wery and Diliberto (2017) compared OpenDyslexic—another DF font—to Arial Regular and TNR Regular and found no improvement in either accuracy or reading speed in children with dyslexia.

This short review of the literature indicates that there is no direct evidence of a facilitatory effect of the DF dedicated graphic features embedded in the letterform, while the facilitatory effect of spacing is controversial. Moreover, no study among those that attempted to test letterform and spacing separately dissociated the spacing variable into its two components: letter spacing and word spacing.

The present study aims to assess the relative contribution of three variables (letterform, inter-letter spacing, inter-word spacing) to reading improvement, by increasing either speed or accuracy, or both, in two groups of children with vs. without dyslexia.

## Methods

### Participants

A total of 128 children participated in the study. Sixty-four children (mean age = 12.4 ± 1 years, 28 females) were with dyslexia (International Classification of Diseases 10 code: F81.0), as certified by healthcare facilities which are a part of the Italian National Health Service, and 64 children (mean age = 12.4 ± 1 years, 25 females) were chronological-age control participants without reading disorders. All children were native Italian speakers. Children with an intellectual disability, attention deficit hyperactivity disorder, neurological diseases, psychiatric disorders, or vision/hearing problems were not included in the study. All children were recruited from regular first grade secondary schools (in the Italian educational system, which corresponds to middle school in the American educational system) in northeast Italy. Procedures were conducted in accordance with the Code of Ethics of the World Medical Association (Declaration of Helsinki), and the study was approved by the local university ethic committee (no. 87, April 11, 2018). Prior to the children’s participation in the study, parents signed an informed consent, granting authorization for their children to participate.

### Materials

Reading material consisted of 9 texts, with each text printed in black on a single white sheet of A4 paper. Text size was 14-point (pt.) as recommended in the British Dyslexia Association guidelines (2012), with line spacing set at 22 pt., and the texts were left-justified in order to keep the within-text inter-word spacing constant. The first text was a baseline text, printed in the Comic Sans MS font with default inter-letter and inter-word spacing. The subsequent 8 texts were test texts, which were comparable according to several psycholinguistic variables (see Appendix 1 in the Electronic Supplementary Material (ESM)), and were presented to children in 8 different conditions.

Two fonts were used: standard font and dyslexia-friendly (DF) font. The standard font was redesigned to be very similar in shape and mathematical proportions to the Verdana Pro Condensed font, a sans-serif font of the Verdana typeface family. The DF font was created ad hoc for the study by adding “dyslexia-friendly” features (specific letterform and spacing) to the standard font (see Appendix 2 in the ESM); in particular, the resulting font was very similar to EasyReading^™^, an Italian font designed for individuals with dyslexia, used in the experiment by Bachmann (2013). The typographical variables—letterform, inter-letter spacing, and inter-word spacing—were manipulated between text as follows.

#### Letterform

In the standard font, letters have a similar or identical shape when rotated relative to the vertical or horizontal axis (e.g., “b-d-p-q” group; Fisher, Liberman, & Shankw, 1978). DF font used asymmetric shapes (e.g., vertical or horizontal asymmetry in the “b-d-p-q” group), mixed serif and sans serif letters, and longer ascenders/descenders, in order to make each letterform distinctive.

#### Inter-letter and inter-word spacing

These parameters varied independently along two levels: default vs. increased spacing. When set to default, the inter-letter and inter-word spacing were equal to those of the Verdana Pro Condensed font. The increased spacing was obtained by enlarging the default spacing: the inter-letter spacing was increased by + 70 1/1000 em, i.e., 0.98 pt. on a body size of 14 pt., while the inter-word spacing by + 270 1/1000 em, i.e., 3.78 pt. on a body size of 14 pt.

**Fig. 1:**
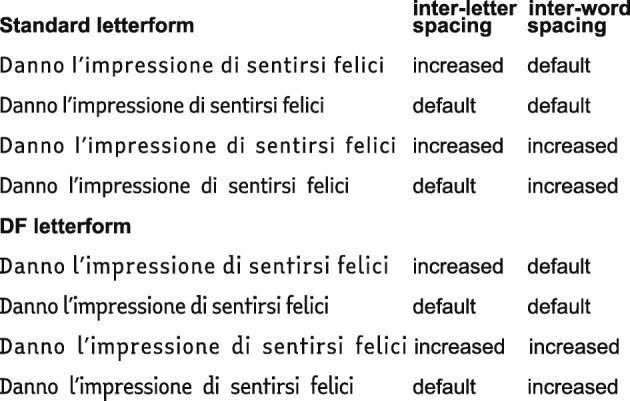
An example of a brief sentence used in one of the test text (English translation: “They give the impression of being happy”), presented in the 8 different conditions in which the reading test material was administrated

The 2 (letterform) × 2 (inter-letter spacing) × 2 (inter-word spacing) experimental design was defined by 8 conditions, as illustrated in Fig. 1.

### Procedure

The experiment took place in a quiet room in the school attended by the children. The reading material was administered individually to the children, and each experimental session lasted approximately 40 min. Participants were asked to start reading each text aloud as soon as the experimenter provided them a verbal signal (“go”), and the reading sessions were audio-recorded by means of a digital recording device. Reading distance was relatively constant across participants (33 ± 3 cm). Five-minute pauses were included after the second, fourth, and sixth texts.

Every participant was required to read the baseline text at the beginning of the experimental session, and the 8 different test texts consecutively, each one written using a different typographical condition (see Fig. 1). The repetition of the same text across the 8 conditions was avoided in order to prevent learning effects. Following the same procedure used by Marinus et al. (2016) to combine texts and conditions, we generated two Latin square matrices, one for the texts (12345678, 23456781, 34567812, 45678123, 56781234, 67812345, 78123456, 81234567) and a second one for the conditions (ADEHCBGF, BCFGDAHE, GFCBEHAD, HEDAFGBC, EBGDAFCH, FAHCBEDG, CHAFGDEB, DGBEHCFA). This resulted in 64 (8 × 8) different text and condition combinations, each one randomly assigned to a child. Following this procedure, overall, every text was presented 8 times in every condition to avoid confusion between texts and conditions.

## Results

Reading speed and accuracy were evaluated for the baseline text and each of the 8 test texts. Reading speed was calculated as the number of syllables per second, with the total duration of reading (in seconds) recorded by the experimenter from the “go” signal given to the participant until the end of his/her reading. Accuracy was computed as the logarithm of the number of words read correctly divided by the number of errors; an error was counted when the participant inaccurately read a word (regardless of the type of error and the number of mistaken letters). The distributions of the two dependent measures in the experimental conditions were analyzed separately. All analyses were performed using the open software Jamovi (version 0.9.5.12; The jamovi project, 2019). The General Analyses for Linear Models in the Jamovi 1.0.1 module was used to generate the mixed effects models.

Data regarding the reading performance of children with dyslexia and the participants in the chronological-age control group in the baseline text were analyzed first. As expected, the analysis of variance (ANOVA) with group (dyslexics, controls) as a between-subjects factor revealed a significant difference between the two groups on both reading speed and accuracy. Dyslexics’ reading speed (*M* = 2.91 syll/s, *SD* = 0.82) was approximately 35% lower than that of controls (*M* = 4.49 syll/s, *SD* = 0.69) (*F*(1,122) = 139, *p* < 0.001, *η*_*p*_^2^ = 0.533). Moreover, dyslexics’ accuracy level (*M* = 1.50, *SD* = 0.33) was approximately 27.5% lower than that of controls (*M* = 1.82, *SD* = 0.28) (*F*(1,122) = 34.9, *p* < 0.001, *η*_*p*_^2^ = 0.222).

Secondly, the performances of the two groups with test texts were analyzed. The average reading speed and accuracy achieved by individuals with dyslexia and typical readers (the control group) are reported separately for the 8 conditions in Table 1. Data were analyzed by means of two separate mixed effects models employed to test the effects of group, letterform, inter-letter spacing, and inter-word spacing on reading speed and accuracy. The level of significance was set at *p* < 0.05; Bonferroni corrections were applied in case of multiple comparisons.

**Table 1:** Mean scores of reading speed and reading accuracy obtained in the 8 test text conditions. Standard deviations are in parenthesis. Reading speed was computed as syllables per seconds, while reading accuracy as the logarithm of the number of words read correctly divided by the number of errors

			Dyslexics		Controls	
Font	Inter-letter spacing	Inter-word spacing	Speed	Accuracy	Speed	Accuracy
Standard font						
	Increased	Default	3.06 (0.91)	1.59 (0.38)	4.90 (0.73)	1.94 (0.34)
	Default	Default	3.25 (0.88)	1.57 (0.35)	4.94 (0.72)	1.92 (0.30)
	Increased	Increased	3.17 (0.92)	1.59 (0.31)	4.99 (0.69)	1.96 (0.31)
	Default	Increased	3.25 (0.91)	1.52 (0.35)	5.01 (0.74)	1.92 (0.32)
Dyslexia friendly (DF)						
	Increased	Default	3.02 (0.97)	1.53 (0.37)	4.86 (0.77)	1.92 (0.32)
	Default	Default	3.19 (0.91)	1.57 (0.35)	5.03 (0.64)	1.95 (0.30)
	Increased	Increased	3.28 (0.96)	1.61 (0.37)	5.02 (0.67)	1.94 (0.30)
	Default	Increased	3.25 (0.97)	1.61 (0.33)	5.02 (0.64)	1.98 (0.26)

In the linear mixed effects model on reading speed, group (dyslexics, controls), letterform (standard, DF), inter-letter spacing (default, increased), inter-word spacing (default, increased), and their interactions were fixed effects; participant (*N* = 64) and text (8) were included in the model as random intercepts. A significant main effect of group was found (*F*(1,126) = 176.337, *p* < 0.001, *η*_*p*_^2^ = 0.583), indicating that dyslexics (*M* = 3.18 syll/s, *SD* = 0.93) read significantly slower than controls (*M* = 4.97 syll/s, *SD* = 0.70). The model did not show a main effect of letterform (*F*(1,875) = 0.496, *p* = 0.482). We found a significant main effect of inter-letter spacing (*F*(1,875) = 19.797, *p* < 0.001, *η*_*p*_^2^ = 0.022) with default spacing read faster (*M* = 4.12 syll/s, *SD* = 1.20) than increased spacing (*M* = 4.04 syll/s, *SD* = 1.23); and a significant main effect of inter-word spacing (*F*(1,875) = 25.624, *p* < 0.001, *η*_*p*_^2^ = 0.028) with default spacing read slower (*M* = 4.03 syll/s, *SD* = 1.22) than increased spacing (*M* = 4.12 syll/s, *SD* = 1.21). As for the interactions, we found a significant effect of inter-letter spacing × inter-word spacing (*F*(1,875) = 12.751, *p* < 0.001, *η*_*p*_^2^ = 0.014). This interaction showed that (a) texts presented in the condition with default inter-letter spacing and default inter-word spacing (*M* = 4.10 syll/s, *SD* = 1.19) were read faster than texts presented in the condition with increased inter-letter spacing and default inter-word spacing (*M* = 3.96 syll/s, *SD* = 1.25, *t*(127) = − 4.803, *p*_bonferroni_ < 0.001, *d =* − 0.424); (b) texts presented in the condition with increased inter-letter spacing and increased inter-word spacing (*M* = 4.11 syll/s, *SD* = 1.21) were read faster than texts presented in the condition with increased inter-letter spacing and default inter-word spacing (*t*(127) = − 5.328, *p*_bonferroni_ < 0.001, *d* = − 0.471); (c) texts presented in the condition with default inter-letter spacing and increased inter-word spacing (*M* = 4.13 syll/s, *SD* = 1.21) were read faster than texts presented in the condition with increased inter-letter spacing and default inter-word spacing (*t*(127) = − 5.859, *p*_bonferroni_ < 0.001, *d* = − 0.518). The other comparisons revealed no significant effects.

Additionally, performances were analyzed with a second linear mixed effects model on reading accuracy: group (dyslexics, controls), letterform (standard, DF), inter-letter spacing (default, increased), inter-word spacing (default, increased), and their interactions were integrated into the model as fixed effects, while participant and text were integrated as random intercepts. Results showed a main effect of group, (*F*(1,126) = 176.337, *p* < 0.001, *η*_*p*_^2^ = 0.583), demonstrating that dyslexics were less accurate (*M* = 1.57 syll/s, *SD* = 0.35) than controls (*M* = 1.94 syll/s, *SD* = 0.31). Despite the model revealing a significant letterform × inter-letter spacing interaction, (*F*(1,875) = 4.327, *p* = 0.038, *η*_*p*_^2^ = 0.004), pairwise post hoc comparisons (Bonferroni-corrected *t* tests) showed no significant effects. The other main effects and interactions tested with the model did not demonstrate statistical significance.

Since in both analyses (with reading speed and accuracy as dependent measures, respectively) the factor group did not interact with the other fixed effects (letterform, inter-letter spacing, inter-word spacing, and their interactions), the performances obtained in the test texts by the two groups (dyslexics and controls) were also analyzed separately.

### Dyslexic group

**Fig. 2:**
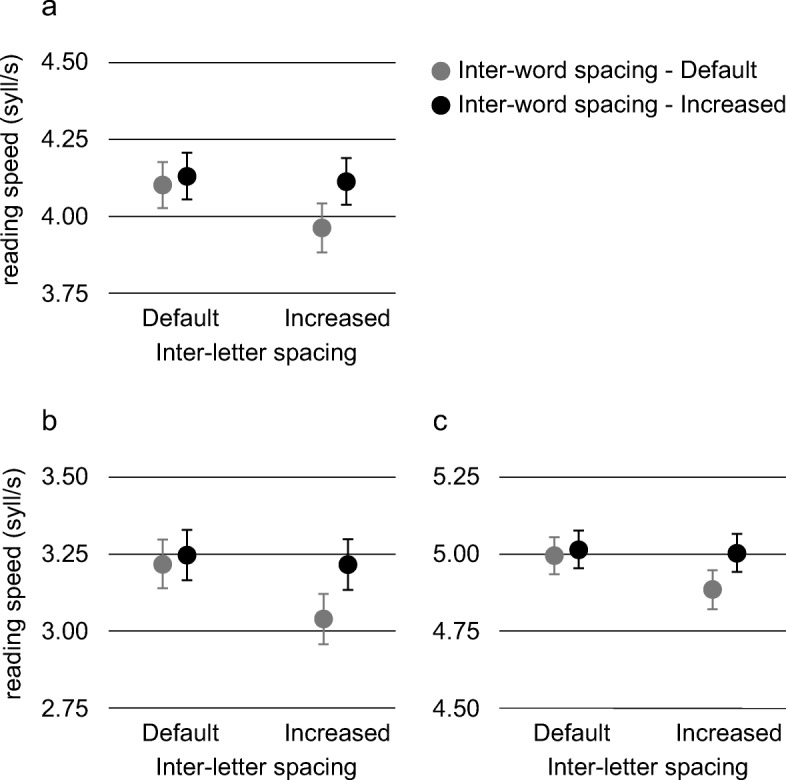
Interaction between inter-letter spacing and inter-word spacing obtained in the general analysis (panel **a**), in the analysis of dyslexics (panel **b**), and in the analysis of controls (panel **c**). Mean scores of reading speed (syll/s) in the 4 spacing conditions (error bars represent ± 1 standard error of mean). Spacing conditions with a default inter-word spacing are depicted in gray, while spacing conditions with an increased inter-word spacing are depicted in black

In the first linear mixed effects model on reading speed, letterform (standard, DF), inter-letter spacing (default, increased), inter-word spacing (default, increased), and their interactions were included as fixed effects, while participant and text as random intercepts. The model did not show a main effect of letterform (*F*(1,434) = 0.006, *p* = 0.936). We found a significant main effect of inter-letter spacing (*F*(1,434) = 15.710, *p* < 0.001, *η*_*p*_^2^ = 0.034) with default spacing read faster (*M* = 3.23 syll/s, *SD* = 0.91) than increased spacing (*M* = 3.13 syll/s, *SD* = 0.94), and a significant main effect of inter-word spacing (*F*(1,434) = 17.059, *p* < 0.001, *η*_*p*_^2^ = 0.037) with default spacing (*M* = 3.13 syll/s, *SD* = 0.92) slower to read than increased spacing (*M* = 3.23 syll/s, *SD* = 0.94). As for the interactions, we found a significant effect of inter-letter spacing × inter-word spacing (*F*(1,434) = 8.984, *p* = 0.003, *η*_*p*_^2^ = 0.020) (see Fig. 2b). This interaction showed that (a) texts presented in the condition with default inter-letter spacing and default inter-word spacing (*M* = 3.22 syll/s, *SD* = 0.90) were read faster than texts presented in the condition with increased inter-letter spacing and default inter-word spacing (*M* = 3.04 syll/s, *SD* = 0.94) (*t*(63) = − 4.258, *p*_bonferroni_ < 0.001, *d* = − 0.532; (b) texts presented in the condition with increased inter-letter spacing and increased inter-word spacing (*M* = 3.23 syll/s, *SD* = 0.94) were read faster than texts presented in the condition with increased inter-letter spacing and default inter-word spacing (*t*(63) = − 3.981, *p*_bonferroni_ < 0.001, *d* = − 0.498; (c) texts presented in the condition with default inter-letter spacing and increased inter-word spacing (*M* = 3.25 syll/s, *SD* = 0.94) were read faster than texts presented in the condition with increased inter-letter spacing and default inter-word spacing (*t*(63) = − 4.727, *p*_bonferroni_ < 0.001; *d* = − 0.591). The other comparisons revealed no significant effects. These results are consistent with a significant impairment of reading speed in the spacing condition less favorable to word segmentation, i.e., the condition with increased inter-letter spacing and default inter-word spacing.

**Fig. 3:**
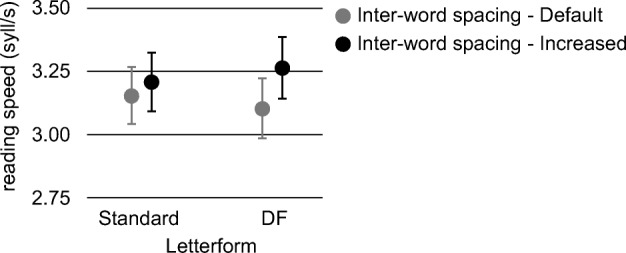
Dyslexics: interaction between letterform and inter-word spacing. Mean scores of reading speed (syll/s) in the 4 conditions defined by letterform (standard, DF) and inter-word spacing (default, increased) (error bars represent ± 1 standard error of mean). Conditions with a default inter-word spacing are depicted in gray, while conditions with an increased inter-word spacing are depicted in black

Moreover, even the interaction letterform × inter-word spacing was significant (*F*(1,434) = 4.471, *p* = 0.035, *η*_*p*_^2^ = 0.010) (see Fig. 3). With the text printed in the DF letterform, the reading speed improved significantly when we enlarged the inter-word spacing from the default (*M* = 3.10 syll/s, *SD* = 0.94) to the increased spacing level (*M* = 3.26 syll/s, *SD* = 0.96) (*t*(63) = 3.33, *p*_bonferroni_ < 0.001, *d* = 0.417). A similar inter-word spacing enlargement did not produce significant effects with the text printed in the standard letterform (*t*(63) = − 1.23, *p*_bonferroni_ = 0.223, *d* = − 0.154) (condition with standard letterform and default inter-word spacing: *M* = 3.16 syll/s, *SD* = 0.90; condition with standard letterform and increased inter-word spacing: *M* = 3.21 syll/s, *SD* = 0.92). Finally, reading speed was not affected by the letterform when both the standard and the DF letterform were presented with an increased inter-word spacing (*t*(434) = 1.22, *p*_bonferroni_ = 0.228, *d* = 0.153).

The second linear mixed effects model on reading accuracy with letterform (standard, DF), inter-letter spacing (default, increased), inter-word spacing (default, increased), and their interactions included as fixed effects, and participant and text as random intercepts, showed no significant main or interaction effects.

### Typical readers group (controls)

In the first linear mixed effects model on reading speed, letterform (standard, DF), inter-letter spacing (default, increased), inter-word spacing (default, increased), and their interactions were included as fixed effects, while participant and text were included as random intercepts. The reading speed of the typical readers was not influenced by the letterform (*F*(1,434) = 0.843, *p* = 0.359). We found a significant main effect of inter-letter spacing (*F*(1,434) = 5.475, *p* = 0.020, *η*_*p*_^2^ = 0.012) with default spacing read faster (*M* = 5.00 syll/s, *SD* = 0.69) than increased spacing (*M* = 4.94 syll/s, *SD* = 0.72), and of inter-word spacing (*F*(1,434) = 9.251, *p* = 0.002, *η*_*p*_^2^ = 0.021) with default spacing read slower (*M* = 4.93 syll/s, *SD* = 0.71) than increased spacing (*M* = 5.01 syll/s, *SD* = 0.68). As for the interactions, we found a significant effect of inter-letter spacing × inter-word spacing (*F*(1,434) = 4.251, *p* = 0.040, *η*_*p*_^2^ = 0.010) (see Fig. 2c). This interaction showed that (a) texts presented in the condition with default inter-letter spacing and default inter-word spacing (*M* = 4.99 syll/s, *SD* = 0.68) were read faster than texts presented in the condition with increased inter-letter spacing and default inter-word spacing (*M* = 4.88 syll/s, *SD* = 0.75) (*t*(63) = −2.577, *p*_bonferroni_ = 0.036, *d* = − 0.322); (b) texts presented in the condition with increased inter-letter spacing and increased inter-word spacing (*M* = 5.01 syll/s, *SD* = 0.68) were read faster than texts presented in the condition with increased inter-letter spacing and default inter-word spacing (*t*(63) = − 3.544, *p*_bonferroni_ < 0.001, *d* = − 0.443); (c) texts presented in the condition with default inter-letter spacing and increased inter-word spacing (*M* = 5.02 syll/s, *SD* = 0.69) were read faster than texts presented in the condition with increased inter-letter spacing and default inter-word spacing (*t*(63) = − 3.518, *p*_bonferroni_ < 0.001, *d* = − 0.440). The other comparisons revealed no significant effects. In other words, typical readers’ performances in terms of reading speed significantly decreased when presented with a text with an increased inter-letter spacing and a default inter-word spacing.

The second mixed effects model on accuracy, with the same variables as the previous model on reading speed included as fixed effects and random components, did not show significant main or interaction effects.

## Discussion

The present study was conceived to investigate whether a font specifically designed for individuals with dyslexia—which presents increased spacing and letterform with dedicated graphic features—could facilitate reading in both children with dyslexia and chronological age control participants. Moreover, to study in depth the contribution of spacing to improving text legibility, this variable was dissociated into its inter-letter and inter-word components. Both children with dyslexia and typical readers did not show differences in reading performance in terms of reading speed and accuracy when they read texts written with the DF letterform or with the standard letterform. These results could be interpreted as additional evidence (see Kuster et al., 2017; Wery & Diliberto, 2017) that the particular letterform of the dyslexia-friendly fonts, which includes dedicated serifs, longer ascenders/descenders, and asymmetry of characters similar in shape, did not produce any advantage in reading over the letterform of common fonts.

One of the strengths of this study is that the two components of text spacing, inter-letter and inter-word spacing, were investigated independently. Both the inter-letter and inter-word spacing were manipulated along two levels, i.e., default spacing and increased spacing. This design determined that one out of the four spacing conditions was characterized by having the inter-letter spacing increased while the inter-word spacing was set at default: both groups of participants showed a decrease in reading speed when presented with this spacing condition, although the effect size was small. This was an expected result since in this unusual spacing condition, it is difficult to segment sentences into words because the sizes of the (increased) inter-letter and the (default) inter-word spacing appeared similar. However, contrary to several studies (Bachmann, 2013; Marinus et al., 2016; Zorzi et al., 2012), but in line with other previous research (Damiano, Gena, & Venturini, 2019; Kuster et al., 2017), a general advantage in reading speed due to increased spacing, in both its components, was not found either in children with dyslexia or in typical readers. The difference between default and increased spacing employed in the present study was smaller than that used in the study by Zorzi et al. (2012), where the authors, using exceptionally wider spacing, found a positive effect on fluency in Italian and French readers. On the contrary, in our study, the inter-letter and inter-word spacing were enlarged by the same amount as in the study by Bachmann (2013), and were equal to the spacing used in the Italian dyslexia-friendly font EasyReading^™^. One plausible explanation for the discrepancy of these results could be the length of the sentences. In fact, both Zorzi et al. (2012) and Bachmann (2013) compared sentences with default spacing with sentences with increased spacing—that are shorter—and it has been demonstrated that short sentences are easier to read (Schneps et al., 2013). Moreover, this discrepancy in results could also be explained by the difference in the amount of increased spacing (with Zorzi’s results), and the age of participants, that could justify the inconsistency with Bachmann’s results. Here, participants were children who attended first grade secondary schools; Bachmann (2013) tested children who were in their fourth year of elementary school: one hypothesis could be that wider spacing supports a child during reading acquisition, but is irrelevant when they have already acquired reading skills.

Considering the performances of children with dyslexia in terms of reading speed, a significant interaction between letterform and inter-word spacing was found; reading speed did not vary when a text with DF or standard letterform and increased spacing was presented. However, reading speed was reduced when the DF letterform was presented with a default instead of an increased spacing, while the same did not occur for standard letterform. In trying to account for this result, it might be argued that the difference between the standard and DF letterforms, with the latter having longer ascenders/descenders, has been shown to change the perception of the amount of inter-word spacing depending on the letterform in use, despite the spacing being equal between the two conditions (standard vs. DF letterform). In other words, participants might have perceived the default inter-word spacing as narrower when DF letterform was employed than when standard letterform was used, because the ratio between the font body with DF letterform (i.e., the distance between the line of the longer descender and the line of the longer ascender) and the inter-word spacing was greater than the ratio computed for the font with standard letterform (in spite of the two fonts being equalized on the *x*-height).

Despite statistically significant main or interaction effects being found both in the general analysis and in the analyses within each group of participants (dyslexics, controls), the effects involving the spacing variables (inter-letter and inter-word spacing), and the interaction effect between letterform and inter-word spacing, were not practically meaningful (e.g., the difference in reading speed between the two conditions did not exceed 0.20 syll/s).

The data showed no effect of letterform and inter-letter and inter-word spacing on reading accuracy either in children with dyslexia or in the control group. This outcome is not surprising since our participants were native Italian speakers who attended the first year of secondary school, and it has been demonstrated that reading accuracy in transparent languages like Italian reaches the ceiling in skilled readers already by the time children attend elementary school (Seymour, Aro, & Erskine, 2003). Moreover, it has been demonstrated that Italian individuals with dyslexia, despite exhibiting extremely slow reading, present a quite preserved reading accuracy, probably due to their typical nonlexical reading allowed by the Italian shallow writing system (Barca, Burani, Di Filippo, & Zoccolotti, 2006; Tressoldi, Stella, & Faggella, 2001; Zoccolotti et al., 1999).

Changes in reading speed and accuracy are the most used method to measure the efficacy of DF fonts. One can wonder, however, whether dyslexia-friendly fonts, despite not affecting overt behavioral measures, have an impact on comprehension. To our knowledge, there is only one study that investigated the effect of typographical features—the presence of serif—on comprehension (Soleimani & Elham, 2012), and the conclusions were that serifs do not have an impact on comprehension. Reading comprehension was not addressed in this study; however, recent research on the readability of websites in individuals with dyslexia (Damiano et al., 2019) showed that the use of the dyslexia-friendly EasyReading^™^ font did not result in better comprehension of the reading material than a standard font.

## Electronic supplementary material

ESM 1 (DOCX 15 kb)

ESM 2 (PDF 104 kb)

## Footnotes

1 In this article, in line with Kinross (2004), the term *font* is used when referring to files that contain instructions for printing or displaying the characters, and *typeface* when referring to a generic set of fonts which are published under the same name.
