# Supplementary material for: Inter-letter spacing, inter-word spacing, and font with dyslexia-friendly features: testing text readability in people with and without dyslexia
Source: Ann Dyslexia. 2020 Mar 14;70(1):141–52. doi: 10.1007/s11881-020-00194-x (PMC7188700; doi:10.1007/s11881-020-00194-x)
Supplement: Supplementary file 1 — (DOCX 15 kb) [file 11881_2020_194_MOESM1_ESM.docx]

**Appendix 1**

Test texts were derived from 8 excerpts taken from children’s books. The excerpts were then modified in order to make them comparable according to several psycholinguistic variables. Final texts included the same number of words (83) as well as comparable average number of words per sentence and average number of words per line. In each text, 44 or 45 out of the total 83 words were open-class words (i.e., nouns, verbs, adjectives, adverbs), and the remaining words were closed-class words (i.e., articles, pronouns, conjunctions, prepositions, etc.). Open-class words were never repeated within the same text and across texts. Moreover, texts were equivalent for variables like word and lemma frequency (evaluated using CoLFIS, the corpus and frequency lexicon of written Italian; Bertinetto et al. 2005), and average word length in both letters and syllables (see Table 1).

**Table** – Characteristics of the open-class words used in the test texts

|  | **Text 1** | **Text 2** | **Text 3** | **Text 4** | **Text 5** | **Text 6** | **Text 7** | **Text 8** | **F** | ***p*** |
| --- | --- | --- | --- | --- | --- | --- | --- | --- | --- | --- |
| Word frequency | 1605.49  (4240.64) | 1490.98  (4209.11) | 1172.20  (3949.03) | 1283.45  (4032.14) | 1578.53  (5372.67) | 1102.95  (3925.39) | 1441.84  (5360.77) | 1549.11  (5445.98) | .078 | .999 |
| Lemma frequency | 6209.80  (15574.68) | 6395.91  (15640.73) | 4212.07  (11556.44) | 4480.23  (11604.66) | 4602.36  (13798.09) | 2792.95  (8550.13) | 3385.84  (11531.10) | 4243.36  (13883.71) | .409 | .897 |
| Length in letters | 6.36  (2.47) | 6.31  (2.38) | 6.18  (2.33) | 6.39  (2.47) | 6.31  (2.40) | 6.39  (2.40) | 6.39  (2.37) | 6.48  (2.46) | .058 | 1.00 |
| Length in syllables | 2.71  (0.94) | 2.64  (0.93) | 2.60  (0.91) | 2.66  (0.96) | 2.64  (0.93) | 2.70  (0.95) | 2.75  (0.94) | 2.75  (0.97) | .147 | .994 |

Means (and standard deviations) of four psycholinguistic word variables (word frequency, lemma frequency, length in letters and length in syllables) are reported for each one of the 8 test texts. In the second to last column, the F values, resulting from the Analysis of Variance on the psycholinguistic variables with Text as within-subjects factor, are reported, as well as, in the last column, the corresponding *p*-values. Test texts did not differ significantly in any psycholinguistic variable.

No open-class words with hiatus sequences or diphthongs were included in the texts, which were comparable also for the number of open-class words with double consonants (n = 11-12), the number of open-class words with grapheme-to-phoneme conversion rules (n = 15 ± 1), the number of morphologically complex open-class words (n = 5-6), and open-class word’s type of stress (paroxytone words: n = 35 ± 1; proparoxytone words: n = 5; oxytone words: n = 1).
