# Supplementary material for: Inter-letter spacing, inter-word spacing, and font with dyslexia-friendly features: testing text readability in people with and without dyslexia
Source: Ann Dyslexia. 2020 Mar 14;70(1):141–52. doi: 10.1007/s11881-020-00194-x (PMC7188700; doi:10.1007/s11881-020-00194-x)
Supplement: Supplementary file 2 — (PDF 104 kb) [file 11881_2020_194_MOESM2_ESM.pdf]

Comparison between
**Standard font**
**and**
**Dyslexia Friendly (DF) font:**
**Dyslexia friendly features**


Bernardis, P., Galliussi J.,
Gerbino, W.,
Università di Trieste,

Perondi, L., IUAV
Venezia,

Chia, G., ChiaLab
Bologna


**Ascenders and descenders**
DF font has longer ascenders
and descenders

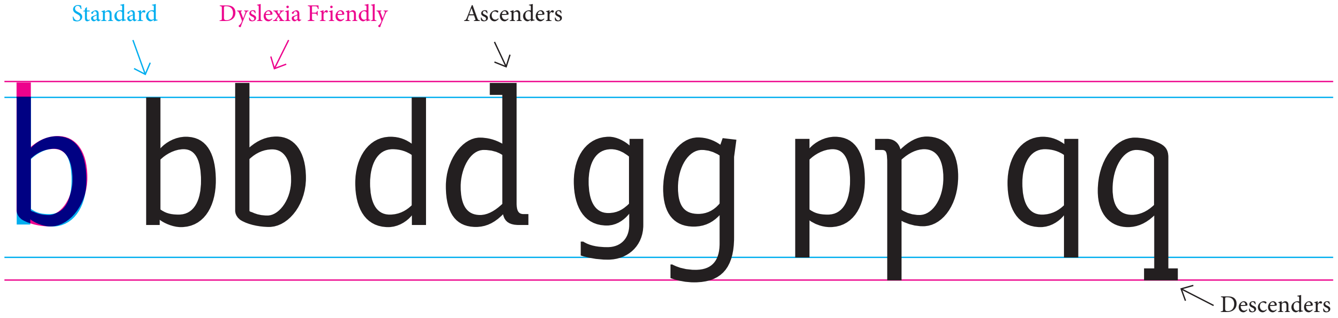


**Asymmetry in letter shapes**
The bows of Stanard font are symmetrical,
while the ones of DF font are asymmetrical

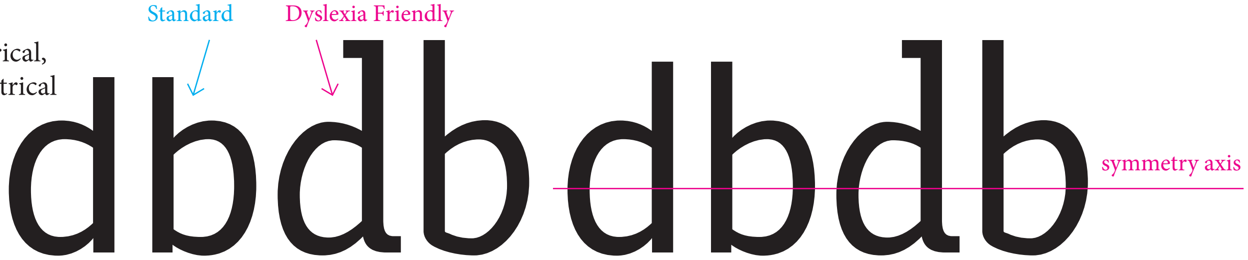


**Dedicated serifs**
Standard font has not serif, while DF font has

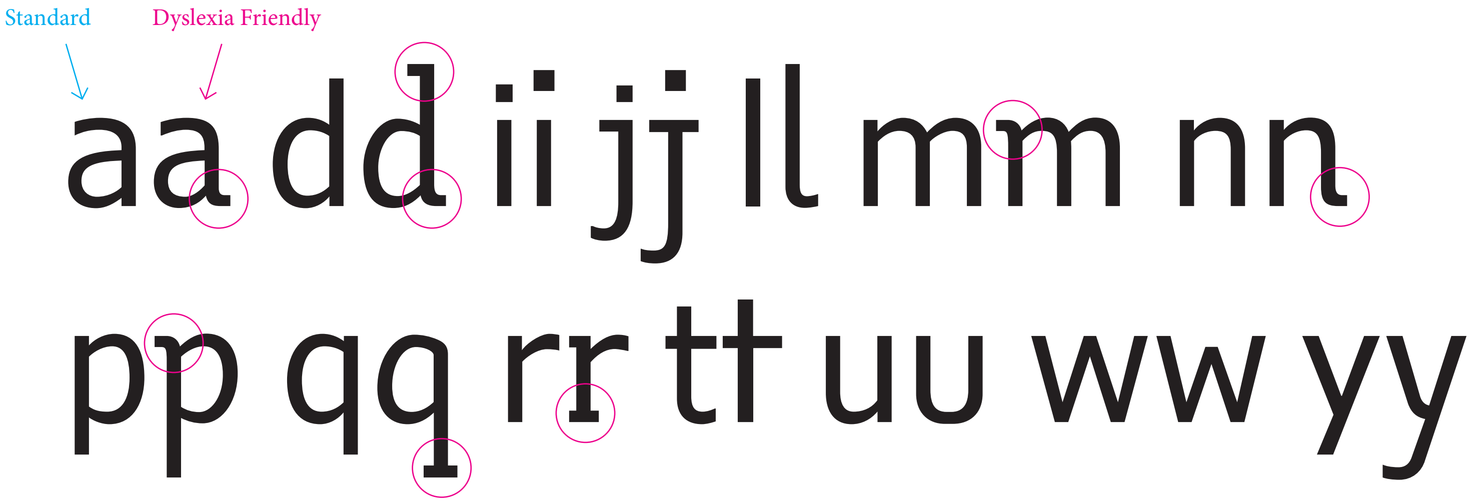

Comparison between
**Standard font**
**and**
**Dyslexia Friendly (DF) font:**
**Spacing and stems**

Bernardis, P., Galliussi J.,
Gerbino, W.,
Università di Trieste,

Perondi, L., IUAV
Venezia,

Chia, G., ChiaLab
Bologna

**Stem thickness**
DF font and Standard font
has the same stem thickness

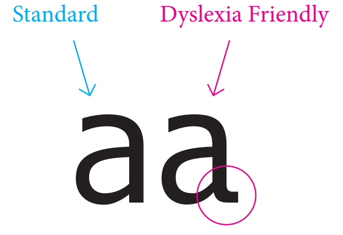


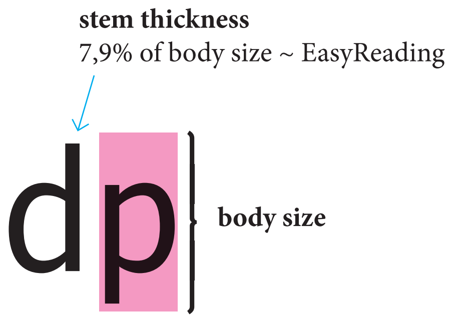


**Inter letter spacing**
Increased by 26%, i.e. + 70 1/1000 em, i.e. 0,98 pt on 14 pt body size

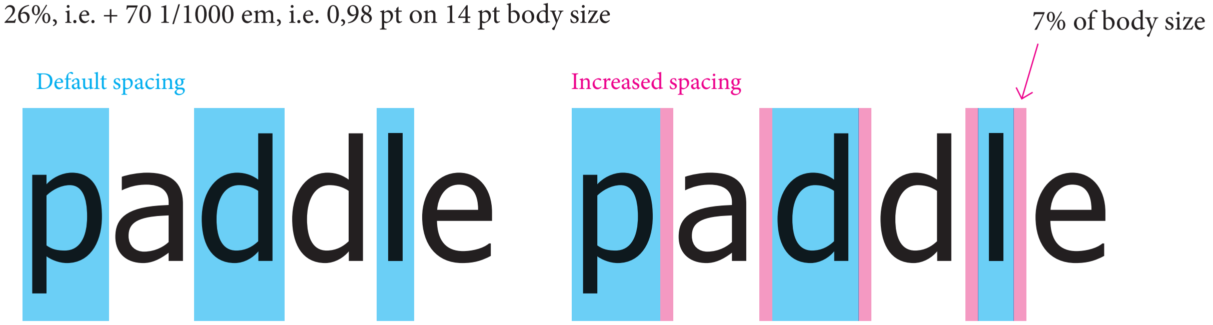


**Inter word spacing**
The inter-word spacing is increased by 100%, i.e. + 270 1/1000 em, i.e. 3,78 pt on 14 pt body size

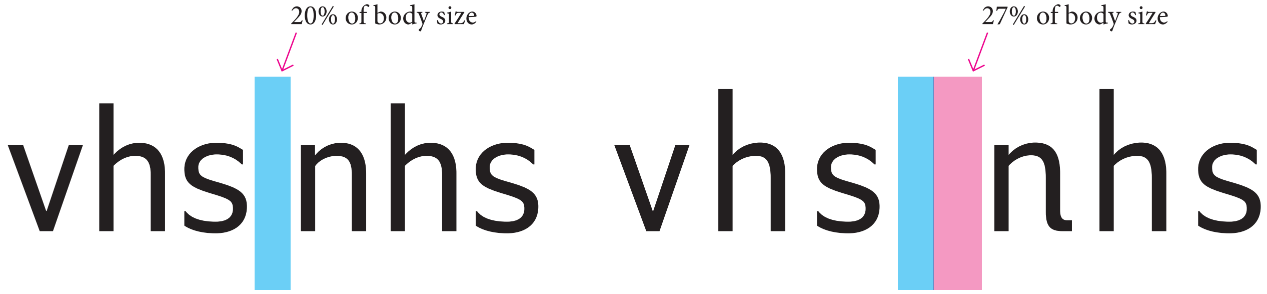

Comparison between
**Standard font**
**Dyslexia Friendly (DF) font**
**Verdana Condensed**
**EasyReading®**

Bernardis, P., Galliussi J.,
Gerbino, W.,
Università di Trieste,

Perondi, L., IUAV
Venezia,

Chia, G., ChiaLab
Bologna

**Letter Shape**
Standard font has approximately the same letter shape of Verdana Condensed

**Ascenders and descenders**
Standard font has the same ascenders as Verdana Condesed
DF font has the same ascenders as EasyReading®

Standard

dislessia

Verdana Condensed

dislessia

Dyslexia Friendly

dislessia

EasyReading®

dislessia

**Stem thickness**
DF font and Standard font has approximately the same stem thickness as EasyReading®

**Dyslexia friendly features**
DF font has approximately the same Dyslexia friendly features as EasyReading®

**Spacing**
DF font and Standard font has been spaced by Igino Marini with iKern

Comparison between
**Standard font**
**Dyslexia Friendly (DF) font**
**Verdana Condensed**
**EasyReading®**

Bernardis, P., Galliussi J.,
Gerbino, W.,
Università di Trieste,

Perondi, L., IUAV
Venezia,

Chia, G., ChiaLab
Bologna

**Characteristics of the Standard font and the Dyslexia Friendly (DF) font.**

The Standard font was redesigned to be very similar in shapes and in mathematical proportions to the Verdana Pro Condensed font, a sans-serif typeface of the Verdana typeface family. The DF font was created ad hoc for the study by adding "dyslexia friendly" features to the Standard font, in particular the resulting font was very similar to EasyReading® a font, especially designed for dyslexics, used in the experiment by Bachmann (2013).

The features are: asimmetry in letter shape, proportion between x-height and ascenders/descenders, stem thickness.

In the Verdana Pro Condensed font, letters have a similar or identical shape after reflection relative to a vertical or horizontal axis, in particular the groups "1-I-L", "b-d-p-q" (Fisher, Liberman, & Shankweiler, 1978), "o-e", "u-n-m". DF font used asymmetric shapes based on the font EasyReading®, including for example big dots in "i-j" group, mixed serif and sans serif letters in "u-n-m", "1-I-L" and "b-d-p-q" groups (see appendix), vertical or horizontal asymmetry in "b-d-p-q" groups.

The length of the ascenders/descenders of DF font matches mathematically the proportion between ascenders/descenders and x-height of EasyReading®: ascenders are 66% of x-height and descenders are 65% x-height. In Verdana and Standard font ascenders are 48% of x-height and descenders are 38% x-height.

The stem thickness in EasyReading® and DF font is ~8% of body size (~16% of x-height), the same with Standard font, which is thinner than Verdana (~9% of body size, ~18% of x-height), as stem thickness is never mentioned as a dyslexia friendly feature, DF font and Standard font had to be identical under this aspect.

The resulting font was very similar to EasyReading®, the two fonts showing exactly the same "dyslexia friendly" features. The two fonts used in the experiments, Standard and DF, thus differed only in the absence (Standard font) vs presence (DF font) of the aforementioned dyslexia friendly features.
